# Erzhi pills ameliorate cognitive dysfunction and alter proteomic hippocampus profiles induced by d-galactose and Aβ_1–__40_ injection in ovariectomized Alzheimer’s disease model rats

**DOI:** 10.1080/13880209.2021.1990353

**Published:** 2021-10-21

**Authors:** Yongyan Xie, Bo Yan, Min Hou, Maofu Zhou, Chao Liu, Mengsheng Sun, Kun He, Cong Fang, Yaohui Chen, Liping Huang

**Affiliations:** a School of Pharmacy, Jiangxi University of Chinese Medicine, Nanchang, China; b Clinical Development Department, Shandong Qidu Pharmaceutical Co., Ltd, Zibo, China; c Pharmacy Department, The Second People’s Hospital of Jingdezhen, Jingdezhen, China; d School of Pharmacy, China Pharmaceutical University, Nanjing, China; e School of Chinese Materia Medica, Beijing University of Chinese Medicine, Beijing, China; f Nephrology Department, Jiang Xi Provincial People’s Hospital Affiliated to Nanchang University, Nanchang, China

**Keywords:** Learning, memory, traditional Chinese medicine, PI3K/Akt signalling pathway

## Abstract

**Context:**

Erzhi pills are a classic Chinese medicine prescription, but their effects on Alzheimer’s disease (AD) are not clear.

**Objective:**

The protective effects of Erzhi pills in AD rats and their potential mechanisms were investigated.

**Materials and methods:**

An AD rat model was established by ovariectomy combined with d-galactose and Aβ_1-40_ injection. Rats were randomly divided into five groups: sham-operated, model, oestradiol valerate (0.80 mg/kg), Erzhi pills high-dose (1.50 g/kg), and Erzhi pills low-dose (0.75 g/kg). Learning and memory abilities were evaluated with the Morris water maze test, oestrogen levels with an ELISA kit, and hippocampal neuron morphology and Nissl bodies in the cytoplasm with H&E and Nissl staining. The expression of ERβ, Aβ_1–40_, and p-tau^404^ was determined by immunohistochemistry. Nano LC-LTQ-Orbitrap Proteomics determined potential targets and related signalling pathways. Western blotting was used to detect the expression of the related proteins.

**Results:**

Erzhi pills (1.5, 0.75 g/kg) markedly reduced escape latencies on the MWM, increased numbers of platform crossings, numbers of neurons, Nissl bodies, oestrogen levels (100.18, 43.04 pg/mL), and ERβ-positive cells (57.42, 39.83); Aβ_1–40_ (18.85, 36.83)- and p-tau^404^ (14.42, 29.71)-positive cells were significantly decreased. Proteomics identified more than 100 differentially expressed proteins involved in 48 signalling pathways, five of which are involved in the PI3K/Akt signalling pathway. Western blotting showed decreased expression of GSK3β and Bad, while Akt, PI3K, 14-3-3, Bcl-xl, and Bcl-2 were upregulated.

**Discussion and conclusion:**

Erzhi pills may serve as a potential agent for AD therapeutics by improving learning and memory.

## Introduction

Alzheimer’s disease (AD) is a neurodegenerative disease characterized by cognitive and memory impairments as its main clinical features. Its pathological characteristics are senile plaques formed by the aggregation of β-amyloid (Aβ) protein and neurofibrillary tangles caused by hyperphosphorylation of intracellular tau protein. Alzheimer’s Disease International reported that there are approximately 50 million dementia patients worldwide in 2018, and it is estimated that this number will reach 82 million by 2030 and 152 million by 2050. AD seriously threatens the lives of the elderly (Patterson 2018). Women are potentially more vulnerable to developing AD than men, as the female brain has been found to be inherently more vulnerable to AD pathogenesis (Pike 2017). Female patients with AD show more severe cognitive dysfunction and pathological changes in the brain than male patients (Irvine et al. 2012). In addition, oestrogen deficiency in early menopause has been associated with an increased risk of AD (Bove et al. 2014). These findings suggest that decreased serum oestrogen levels after menopause may be closely related to the development of AD.

At present, the most effective drugs for AD include acetylcholinesterase inhibitors and *N*-methyl-d-aspartic acid (NMDA) receptor antagonists. Although these two kinds of drugs can alleviate the symptoms of AD to some extent, they also have serious side effects such as gastrointestinal reactions, muscle cramps, insomnia, and bradycardia, and do not cure the disease. In addition, oestrogen replacement therapy is commonly used to relieve the symptoms of AD in menopausal women but has been found to increase the risk of endometrial cancer, ovarian cancer, and breast cancer (Sherwin 2006). None of these single-target drugs can effectively treat AD, which may be related to the complicated pathogenesis of the disease. Recently, clinical investigations have revealed that combined drug therapy can play a good therapeutic role in different pathophysiological processes of AD and leads to much better results than single-target drug therapy (Li et al. 2016). Therefore, an increasing number of medical experts are gradually turning their attention to multi-target drug therapy for AD in the course of new drug discoveries. Chinese medicine formulations are characterized by multiple ingredients, multiple pathways these ingredients are involved in, and multiple pathway targets, and can therefore provide the medical scientific community with new ideas for the treatment of AD (Wu et al. 2015).

According to traditional Chinese medicine (TCM) theory, the occurrence of AD is believed to be closely related to kidney deficiency. In the *Huang Di Nei Jing*, the earliest Chinese medical text that is considered the fundamental doctrine for Chinese medicine, it is reported that the kidney is responsible for the function of the brain. If the ‘essence’ of the kidney is fully replenished, then the brain is sufficiently nourished and shows strong memory and good understanding capacity. In contrast, if there is insufficient ‘essence’ in the kidney, the brain will not be sufficiently nourished, which will lead to poor memory and low comprehension abilities. Another classic Chinese medicine book, the *Yi Xue Xin Wu*, written by Cheng Guopeng of the Qing Dynasty in 1732, put forward the theory that ‘the kidney is responsible for wisdom; therefore, kidney deficiency causes lack of wisdom’. Some clinical studies have indeed shown that the TCM syndrome of AD mainly manifests as kidney deficiency (Hao and Xiao 2014).

Erzhi pills are a classic prescription of Chinese medicine originating from the *Fu Shou Jing Fang* in the Ming dynasty, with the effects of nourishing the kidney ‘essence’ and hemostasis, and strengthening muscles and bones (Cai et al. 2011). Erzhi pills are composed of *Ligustrum lucidum* WT Aiton (Oleaceae) (Nv Zhen Zi) and *Eclipta prostrata* Linn (Asteraceae) (Mo Han Lian). Pharmacological research has shown that Erzhi pills provide enhanced immunity and improved intelligence and that they have anti-ageing, anti-inflammatory, antitumor, and various other effects (Cai et al. 2011). It was found that Erzhi pills can improve the learning and memory abilities of d-galactose induced ageing rats, that they have anti-ageing functions, scavenge brain free radicals, and protect neurons (Gao et al. 2010). Erzhi pills can also significantly improve the physical movement coordination, learning, and memory abilities of 1-methyl-4-phenyl-1,2,3,6-tetrahydropyridine (MPTP)-induced Parkinson’s disease mice (Wu et al. 2019). In our previous study, Erzhi pills improved the learning disorders of ovariectomized AD mice by promoting energy metabolism and vesicle transport (Huang et al. 2017, 2018). The active ingredients of Erzhi pills, such as quercetin, geraniol, β-sitosterol, nerol, and eriodictyol, may ameliorate AD, and the mechanism of action of these compounds is closely related to the PI3K/Akt signalling pathway (Huang et al. 2017). At present, research on Erzhi pills is only conducted at the level of overall efficacy, and its anti-AD mechanism has not yet been clarified. It is also currently unclear whether Erzhi pills can ameliorate cognitive dysfunction in AD rats induced by ovariectomy combined with d-galactose and Aβ_1–40_ injection. These limitations greatly hinder clinical applications.

Wilkins and Williams first proposed the concept of proteomics in 1994, which refers to all proteins expressed in a cell or tissue under specific time and space conditions (Pandey and Mann 2000). Proteomics is now a high-throughput, large-scale, and systematic study of protein composition and dynamic changes in expression levels in cells or tissues at an overall level. This research idea coincides with the holistic view of TCM in treating diseases and the characteristics of multi-linkage and multi-target regulation of Chinese medicines. Therefore, proteomics is important in the research of complex Chinese medicine systems. It can comprehensively explore the multi-path and multi-target regulation processes of various active ingredients in Chinese medicines after entering the body, thereby revealing the molecular mechanisms underlying the treatment effects of Chinese medicines.

In view of the above-mentioned limitations, this study used holistic pharmacodynamics combined with proteomics research to explain the pharmacological effect of Erzhi pills on AD and its underlying mechanism. First, a model of AD rats was established by ovariectomy combined with d-galactose and Aβ_1–40_ injection. The Morris water maze (MWM) test was used to assess the effect of Erzhi pills on learning and memory abilities, while an ELISA kit was used to analyze serum oestrogen levels. The morphology of hippocampal neurons was observed by H&E staining, and Nissl staining was used to observe the Nissl bodies in the cytoplasm. The expression of ERβ, Aβ_1–40_, and p-tau^404^-positive cells was determined by immunohistochemistry. Second, proteomics technology was used to explore the differentially expressed proteins in the hippocampal tissue of rats given Erzhi pills, and to explore the key targets and signalling pathways involved in the anti-AD effect of Erzhi pills. This study provides an important experimental basis for the clinical application of Erzhi pills in the prevention and treatment of AD.

## Materials and methods

### Experimental drugs and reagents

Erzhi pills were purchased from Jiangxi Yaodu Zhangshu Pharmaceutical Co., Ltd. (Jiangxi, China). Specnuezhenide (purity > 98%) was obtained from Jiangxi Bencao Tiangong Biotechnology Co., Ltd. (Jiangxi, China). Oestradiol valerate tablets were purchased from the Guangzhou branch of Bayer (Guangzhou, China). The rat ELISA kit was supplied by Andy Gene Biotechnology Co., Ltd. (Beijing, China). The H&E staining kit and Nissl staining kit were obtained from Jiangsu KeyGEN BioTECH Co., Ltd. (Jiangsu, China). The SP Rabbit HRP (DAB) immunohistochemical kit was obtained from Jiangsu Kang Wei Shi Ji Biotechnology Co., Ltd. (Jiangsu, China). dl-Dithiothreitol, RIPA, and PMSF were obtained from Thermo Fisher Scientific Inc. (USA). Aβ_1–40_, d-galactose, iodoacetamide, acetone, formic acid, trifluoroacetic acid, and acetic acid were supplied by Sigma Aldrich Co. (St. Louis, MO, USA). Sequencing trypsin was purchased from Promega Corporation (Madison, WI, USA). Anti Aβ_1–40_, p-tau^404^, GSK3β, p-GSK3β, 14-3-3β/α, Bad, Bcl-xl, and Bcl-2 antibodies were obtained from Abcam Inc. (Cambridge, UK). p-Akt, Akt, p-PI3K, PI3K, and anti-β-actin antibodies were produced by Cell Signalling Technology (Danvers, MA, USA). All other chemicals were of analytical grade.

### Instrument and equipment

High-performance liquid chromatography (Agilent1260, Agilent Technologies Co., Ltd., USA); Stereotaxis apparatus and Flexible skull drill (ZH-blue star B, ZH-RXZ, Anhui Zhenghua Biological Instrument Equipment Co., Ltd., China); Paraffin section machine and Microscope (RM2255, DMI3000B, Leica Microsystems Co., Ltd., Germany); ORBITRAP ELITE mass spectrometer, EASY-nLC1000 liquid phase analyzer and D-37520 high-speed freezing centrifuge (Thermo Fisher Scientific Inc., USA); ELX800 photoabsorbent enzyme marker (BioTek Instrument Co., Ltd., USA); Bio-Rad imaging system (Chemic Doc XR+, Bio-Rad Laboratories Inc., USA).

### Quality control of Erzhi pills

High-performance liquid chromatography (HPLC) was used to detect the content of specnuezhenide in Erzhi pills in order to evaluate their quality. According to the Pharmacopoeia of China (2015), the content of specnuezhenide in qualified Erzhi pills is above 4.0 mg/g. Erzhi pills sample solution and specnuezhenide reference substance were prepared according to the Pharmacopoeia of China (2015). These solutions were filtered using a syringe filter (0.4 µm), and the filtrates were transferred to HPLC analysis. Samples were analyzed on Zorbax Eclipse XDB columns (4.6 × 150 mm^2^, 5 µm, Agilent, Santa Clara, CA, USA). The mobile phase consisted of methanol and water (36:64). Each sample (10 μL) was injected for analysis, and the profile was recorded at an ultraviolet detection wavelength of 224 nm. The theoretical plate number was not less than 7000, according to the peak of specnuezhenide. Three parallel experiments were performed for each sample, and three parallel injections were performed for each sample.

### Animal experiment

Seventy female Sprague-Dawley rats (2–3 months old, 220–250 g) and 20 female and 20 male Kunming mice (25 ± 2 g) were purchased from Hunan SJA Laboratory Animal Co., Ltd. (Hunan, China; Certificate No. SCXK 2013-0004). Rats were housed under controlled room temperature (23 ± 2 °C) and humidity (50%–70%) and a 12-h light/dark cycle. All experimental procedures involving animals and their care were carried out in accordance with the Guide for the Care and Use of Laboratory Animals (Chinese Council on Animal Research and the Guidelines of Animal Care). The experimental protocol was approved by the Experimental Animal Ethics Committee of Jiangxi University of Traditional Chinese Medicine (Animal Ethics Committee No. JZLLSC2017-0074).

In the acute oral toxicity study, mice were divided into Erzhi pill (60 g/kg) and control groups (vehicle) (*n* = 10/sex/group). General behaviour and mortality were observed after 14 days. The protocol was based on guidelines for acute toxicity studies of traditional Chinese medicine and natural medicine.

Rats were anesthetized with intraperitoneal injections of 10% chloral hydrate (3.0 mL/kg). Bilateral ovariectomy (Huang et al. 2018) was performed using sterile surgical techniques. A small incision was made in the region between the hip and the last rib on each abdominal side. The fallopian tubes were clamped and ligated, and the ovaries and fat tissue around the ovaries were excised. The muscle and skin layers were then sutured. Sham surgery was performed in the same way but without ligation of the fallopian tubes and resection of the ovaries. After surgery, all rats were injected intramuscularly with penicillin sodium (50,000 U, for 3 days) to fight infection. All rats were fed a soy-free diet to exclude the effects of phytosterols in the diet. Two days after surgery, the exfoliated vaginal epithelial cell smear technique was used to evaluate the success of the ovariectomy. If keratinocytes were not detected in five consecutive day smears, which suggested successful ovariectomy, ovariectomized rats were randomly divided into four groups (*n* = 13 per group), labelled the ‘model’, ‘estradiol valerate’, ‘Erzhi pills high-dose’, and ‘Erzhi pills low-dose’ groups. In addition, sham-operated rats were selected for the sham-operated group (*n* = 13).

**Figure 1.**
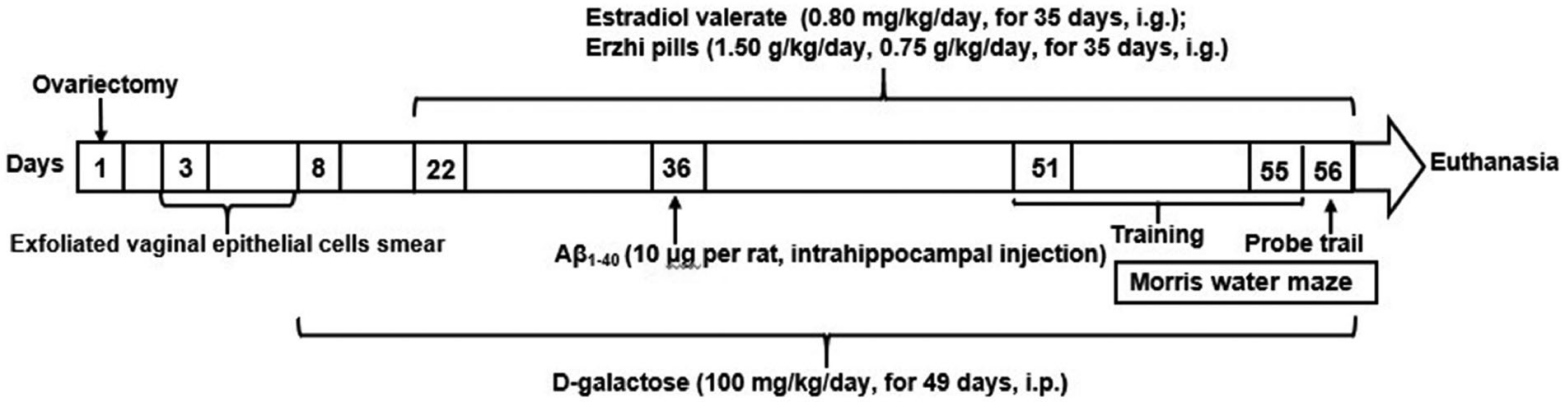
Schematic representation of the animal experimental design.

Except for the sham-operated group, rats were injected intraperitoneally with d-galactose (100 mg/kg/day, for 49 days) on day 8, and they were administered an intrahippocampal injection of Aβ_1–40_ (10 μg per rat) on day 36, while rats in the sham-operated group received equal volumes of saline. On day 22, rats in the oestradiol valerate group were orally treated with oestradiol valerate (0.80 mg/kg/day), while the Erzhi pills high- and low-dose groups were orally treated with Erzhi pills (1.50, 0.75 g/kg/day) for 35 days; rats in the sham-operated and model groups received equal volumes of saline. The MWM test was performed on days 51–55, and the probe trial was performed on day 56. After the MWM test, the blood samples of all rats were collected after anaesthesia with 10% chloral hydrate (3.0 mL/kg, i.p.) and euthanized by cervical dislocation under anaesthesia. Brain samples were harvested. A schematic representation of the experimental animal design is shown in Figure 1.

Aβ_1–40_ injection was performed as previously described, with some modifications (Paxinos and Watson 2005; Souza et al. 2013). Aβ_1–40_ was dissolved in sterile normal saline and incubated at 37 °C for 4 days to obtain the aggregated form before use. All five groups were anesthetized with 10% chloral hydrate (3.0 mL/kg, i.p.). The rats were placed on a stereotaxis apparatus. The bilateral hippocampus and cortex were located (AP = −3.0 mm, ML = 2.0 mm, DV = 2.8 mm from Bregma). For the other four groups, Aβ_1–40_ (5 µL) was injected into the right hippocampus and another 5 µL was injected into the left hippocampus. Sterile normal saline (5 µL each) was slowly injected into the bilateral hippocampus using a micro-syringe for the sham-operated group. The injection lasted for 5 min, and the needle was left for another 2 min to ensure adequate absorption of Aβ_1–40_. After surgery, all rats were injected intramuscularly with penicillin sodium (50,000 U, for 3 days) to prevent infection.

### Morris water maze test

Morris water maze (MWM) test was used to evaluate the learning and memory ability and spatial cognitive function of the rats (Gong et al. 2018). In the MWM test, we used EthoVision XT software (Version 10, Noldus Information Technology b.v., Wageningen, Netherland) to recorded and preliminarily analyze animal behaviour data. MWM contains the oriented navigation test (Training phase) and the spatial probe test (Probe trail). The rats were continuously trained for 5 days, twice a day (AM and PM), and the escape latency was recorded in each group. The time required from entry to swimming on the hidden platform was called the escape latency. Spatial probe test was carried out on the sixth day, the platform was removed, the rats were dropped into the water in a different quadrant and given 60 s to seek for the platform, then the times of crossing platform was recorded in each group, while heatmap was created.

### The determination of serum oestrogen levels

Blood samples were collected from the abdominal aorta after anaesthesia (10% chloral hydrate, 3.0 mL/kg, i.p.), and the samples were centrifuged (4 °C, 3000 rpm, 10 min), then the supernatant was harvested and stored at −20 °C. Oestrogen levels were measured according to the specification of the ELISA kit.

### Morphology of hippocampal neurons

After collected blood samples, rats were rinsed with phosphate-buffered saline (PBS) for cardiac perfusion, and then tissues were fixed in 4% paraformaldehyde. The rats were euthanized by cervical dislocation under anaesthesia (10% chloral hydrate, 3.0 mL/kg, i.p.). Afterward, bilateral hippocampi were quickly excised and were fixed in 10% formaldehyde for 24 h at room temperature, and then embedded in paraffin. The cell morphology of hippocampal neurons was observed according to the specification of the H&E staining kit and Nissl staining kit.

### *The expression of ERβ, Aβ_1_*_–_ *_40_ and p-tau^404^*

The expression of ERβ, Aβ_1-40_ and p-tau^404^ in the hippocampus was determined by immunohistochemistry. Paraffin-embedded hippocampal slices (5 µm) were baked in the oven at 60 °C for 2 h, deparaffinization and rehydration of tissue sections were done by washing in xylene followed by washing in 100%, 95%, and 70% ethanol and rinsing with distilled water. The slices were immersed in the citric acid buffer for antigen retrieval, finally cooled naturally to room temperature. Then the slices were incubated with primary antibodies ERβ (1:400), Aβ_1–40_ (1:100) and p-tau^404^ (1:200) overnight at 4 °C. Cells in 3 fields (200×) were counted in each section under a microscope, and the sum was divided by 3, which was used as the hippocampal cell count of the rats to measure the loss of hippocampal neurons (Huang et al. 2018).

### Proteomics analysis of hippocampal tissues

For the extraction of hippocampal protein, dl-dithiothreitol (DTT) solution was added to the protein samples (*n* = 20, four samples per group), followed by alkylation of iodoacetamide (IAA) solution. The purified protein was precipitated with acetone three times (4 °C, 14,000 × *g*, 10 min), resuspended in ammonium bicarbonate solution, sequenced trypsin was added overnight, and the solution was then desalted with an EASY-Spray Column (C18), followed by vacuum freeze-drying. The samples were centrifuged after redissolution with formic acid (4 °C, 14,000 *g*, 10 min), and the protein supernatant was detected using an LC-MS system (Hou et al. 2016; Huang et al. 2018).

Nanol liquid chromatographic conditions: the analytical column was Easy-Spray column (C18, 3 μm, 100 Å, 75 μm × 15 cm) and the precolumn was Acclaim PepMap^®^ 100 (C18, 3 μm, 100 Å, 75 μm × 2 cm, nanoViper). The injection volume was 2 μL, the flow rate was 250 μL/min, and the column temperature was 20 °C. Mobile phase A was 0.1% formic acid solution, and mobile phase B consisted of 0.1% formic acid acetonitrile solution.

LTQ Orbitrap Elite mass spectrometry conditions: the mass spectrometry condition was set to the positive ion mode, spray voltage was set to 1.9 kV, and the capillary temperature was 250 °C. The first-stage spectrum opens the orbital trap scanning mode of the electrostatic field, and 35% collision energy is generated in the Ion trap mode. The second-stage mass spectrometry information is collected by TOP20 of the Data-dependent Acquisition (DDA) method and Ion trap scanning mode (scanning range: 300 ∼ 1800).

The detection data collected by the LC-MS system were imported into Protein Discovery software for protein identification. SIEVE software was used to perform relative quantitative and qualitative analyses of hippocampal proteins in different groups. Gene ontology (GO) analysis of differential proteins was performed using the PANTHER Classification System database, a network diagram of proteins was constructed from the STRING database, and signal pathways were imported into the Kyoto Encyclopaedia of Genes and Genomes (KEGG) database for analysis.

### Western blotting

After pre-treatment of the rat hippocampal tissue, the total protein of the cells was extracted with the protein extraction kit according to the manufacturer’s instructions, and the protein was quantified using the BCA kit. The protein was denatured by boiling, and SDS-page gel electrophoresis was conducted. The protein was transferred to a PVDF membrane, sealed with 5% BSA for 2 h, and washed three times with TBST. PI3K, p-PI3K (1:1000), Bcl-2 (1:500), Bcl-xl (1:1000), Bad (1:1000), 14-3-3 (1:1000), GSK3β (1:1000), p-GSK3β (1:1000), p-Akt (1:1000), and Akt (1:1000) antibodies were added, respectively, and incubated overnight at 4 °C. After washing three times with TBST, the corresponding secondary antibodies were added and incubated at room temperature for 1 h, and the solution was then washed three times with TBST. Pictures were taken with the Bio-Rad Imager (Bio-Rad Laboratories, Hercules, CA, USA), and protein expression was analyzed using Quantity One software.

### Statistical analysis

Statistical analysis was performed using SPSS 17.0 software, and data were analyzed with analysis of variance (ANOVA) followed by Tukey’s multiple comparison test. In particular, a repeated-measures ANOVA was used to analyze the escape latency in the MWM. Data are expressed as the mean ± SD. Statistical significance was set at *p* < 0.05.

## Results

### Quality control results

Specnuezhenide showed strong absorption under the tested chromatographic conditions, with a good peak and peak shape, stable baseline, and retention time. The average value of specnuezhenide in the samples was 16.00 mg/g (Table 1). This indicates that the Erzhi pills administered in this study fulfil the quality requirements to be used for experimental research.

**Table 1.** The specnuezhenide content of Erzhi pills.

Number of experiment	m_1_ (mg)	Peak area	m_2_ (mg)	Content of Specnuezhenide (mg/g)
One	499.70	1785.80 ± 23.69	7.60	15.20
Two	500.60	1954.70 ± 4.53	8.50	16.90
Three	500.90	1852.20 ± 3.00	8.00	15.90

Note: m_1_: The quality of Erzhi pills powder (precision weighing); m_2_: The quality of specnuezhenide in Erzhi pills.

### Acute toxicity study

**Figure 2.**
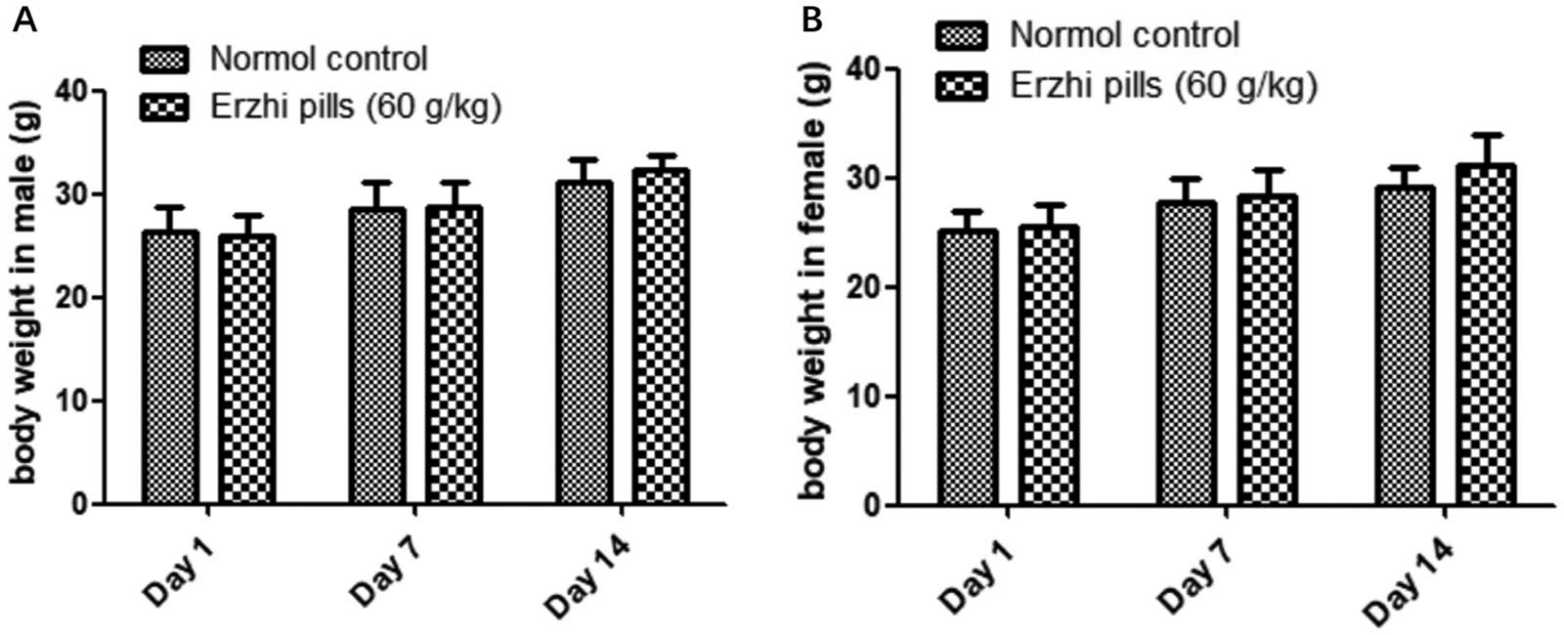
Effects of oral Erzhi pills on body weight in mice. (A) Male mice treated with Erzhi pills in acute toxicity test. (B) Female mice treated with Erzhi pills in acute toxicity test. Data are means ± SD, *N* = 10.

Our acute toxicity studies showed no significant changes in body weight (Figure 2(A,B)) or physiological status. The maximal tolerable dose (MTD) of oral Erzhi pills was 60 g/kg.

### Effects of Erzhi pills on learning and memory abilities and cognitive function in AD rats

**Figure 3.**
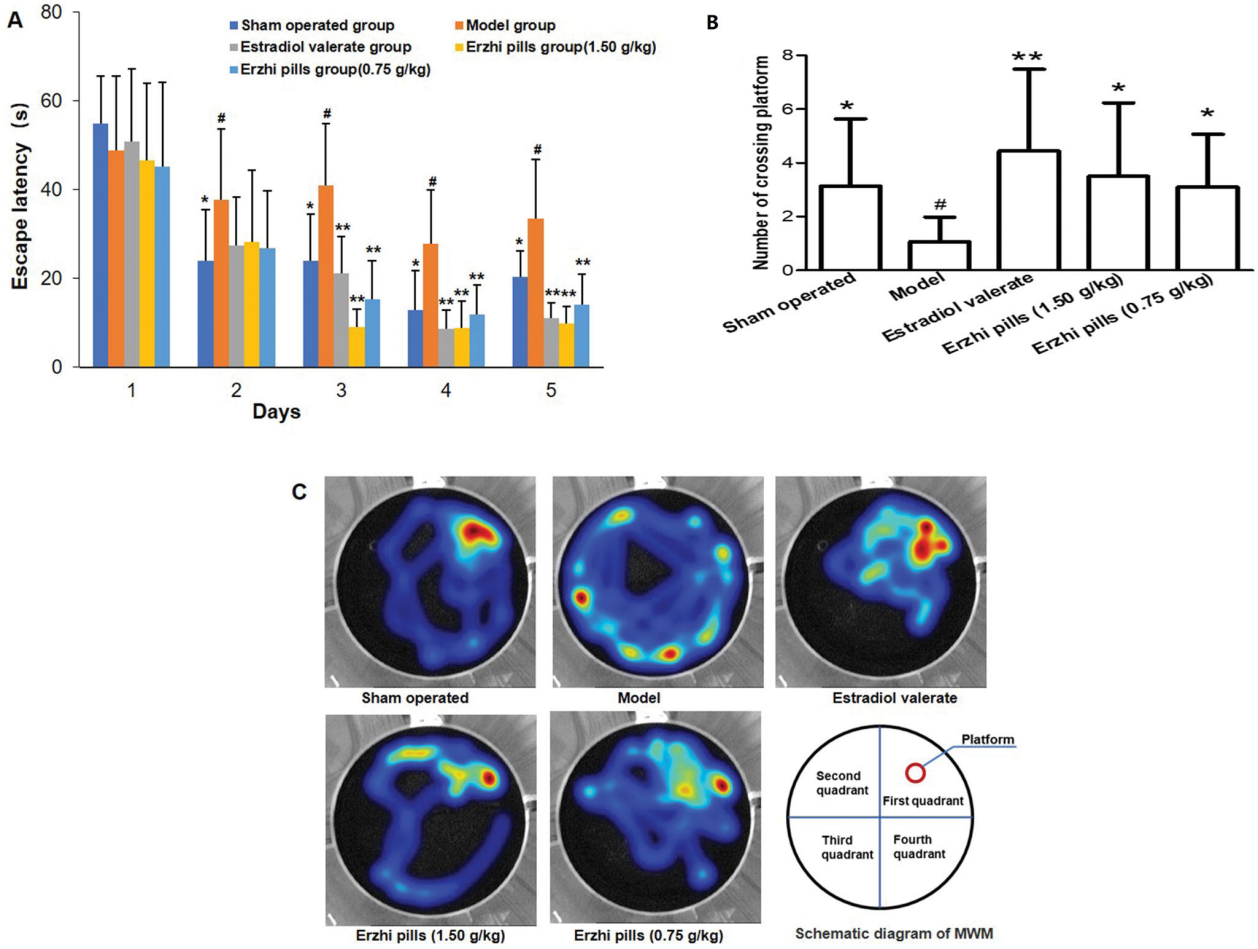
Effect of Erzhi pills on learning and memory ability in AD model rats induced by ovariectomy as well as d-galactose and Aβ_1–40_ injection in Morris water test. (A) The effect of Erzhi pills on the escape latency in AD rats. (B) The effect of Erzhi pills on the times of crossing platform in AD rats. (C)The heatmap of the space exploration experiment in rats. The warmer the hue is in the heatmap, the longer the time will stay. **^#^***p* < 0.05, vs. Sham-operated group; *p < 0.05, **p < 0.01, vs. model group. Mean ± SD, *n* = 10.

The results of the MWM test are shown in Figure 3(A–C). Compared with the sham-operated group, the rats in the model group exhibited longer latencies on days 2, 3, 4, and 5 during the training phase (*p* < 0.05), and their number of platform crossings decreased significantly in the probe trial (*p* < 0.01), suggesting that the rat model of AD was established successfully. Rats in the oestradiol valerate group exhibited shorter latencies on days 3, 4, and 5, compared to the model group, during the training phase (*p* < 0.01). Rats in the high- and low-dose groups exhibited shorter latencies on days 3, 4, and 5 (*p* < 0.01), while their number of platform crossings increased significantly (*p* < 0.05). The stay time in quadrant I (target quadrant) increased in the sham-operated, oestradiol valerate, and Erzhi pill groups compared with the model group (Figure 3(C)).

### Effects of Erzhi pills on oestrogen levels in AD rats

**Figure 4.**
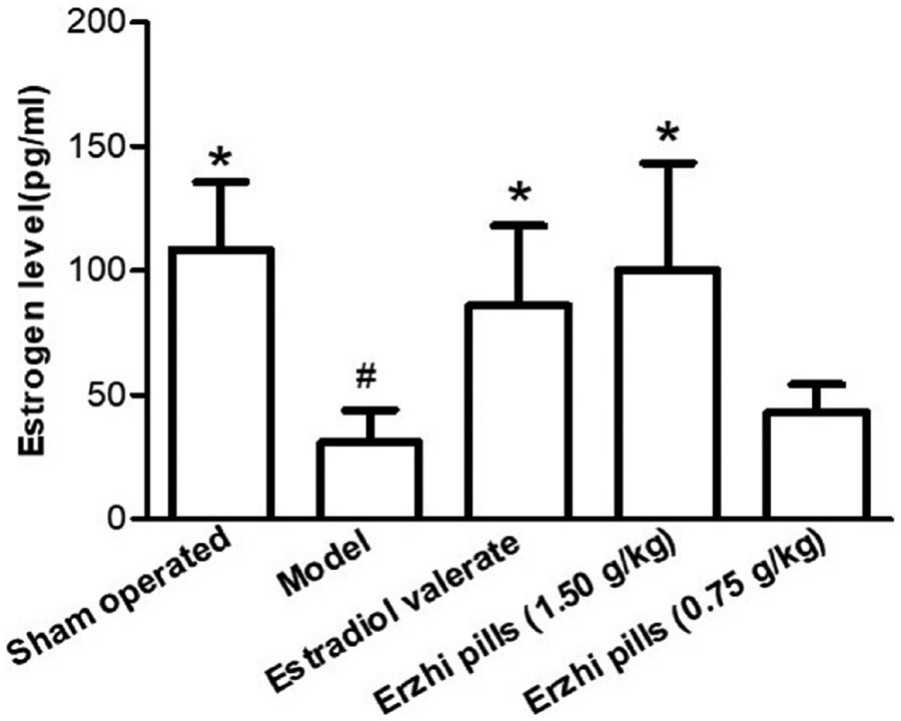
Effect of Erzhi pills on oestrogen level in AD model rats induced by ovariectomy as well as d-galactose and Aβ_1–40_ injection. **^#^***p* < 0.05, vs. Sham-operated group; *p < 0.05, vs. model group. Mean ± SD, *n* = 10.

Results are shown in Figure 4. Compared with the sham-operated group (108.46 ± 27.21 pg/mL), oestrogen levels in the model group decreased (30.79 ± 13.17 pg/mL) (*p <* 0.05), which suggests successful ovariectomy. Oestrogen levels in the oestradiol valerate group (85.9 ± 32.13 pg/mL) and the Erzhi pills high-dose group (100.18 ± 43.29 pg/mL) increased significantly (*p* < 0.05), while there was a decreasing trend in the Erzhi pill low-dose group (43.04 ± 11.29 pg/mL) (*p* > 0.05).

### Effects of Erzhi pills on the morphology and number of hippocampal neurons in AD rats

**Figure 5.**
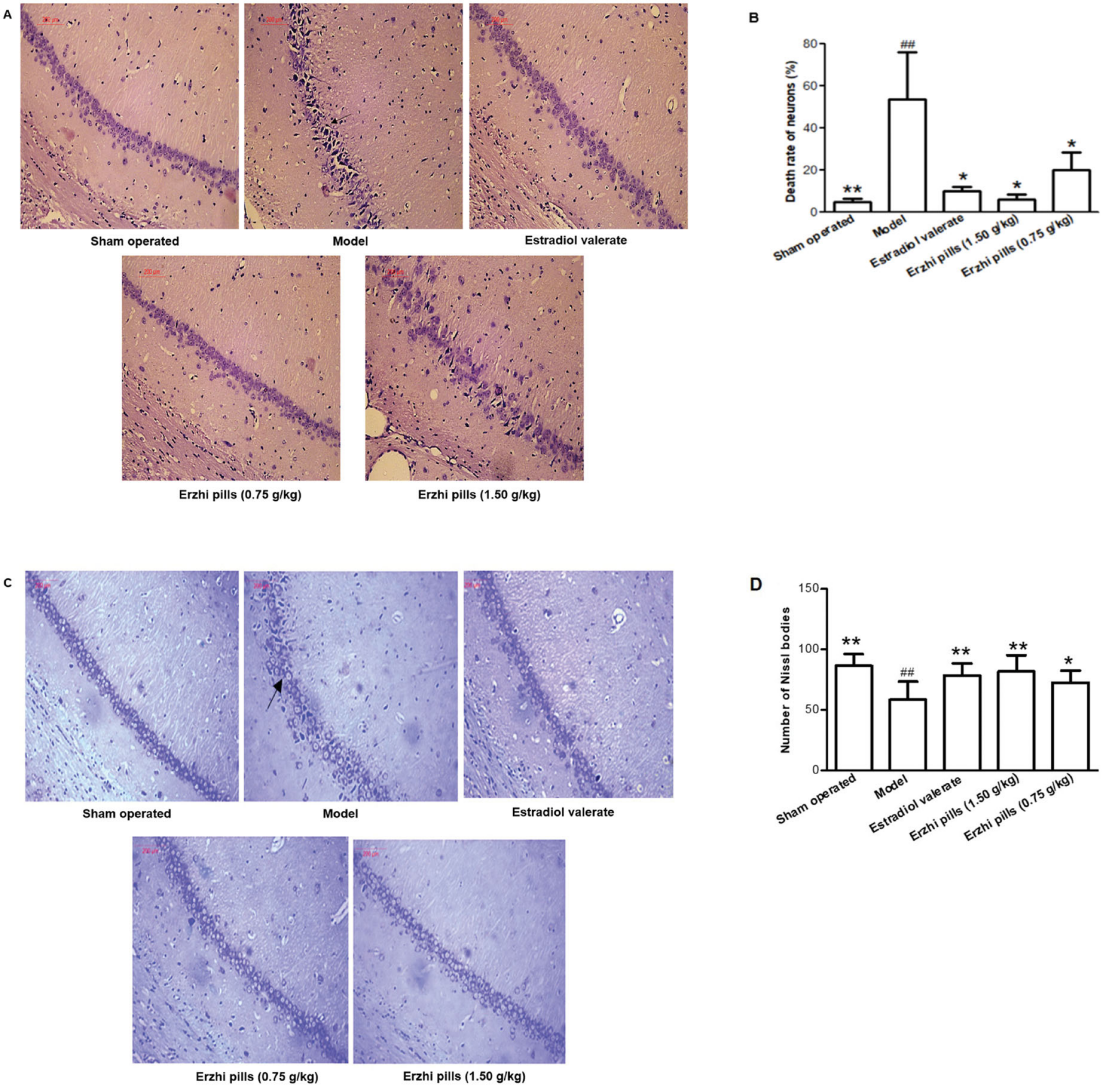
Effect of Erzhi pills on the morphology and number of hippocampal neurons in AD model rats induced by ovariectomy as well as d-galactose and Aβ_1–40_ injection. (A) The effect of the Erzhi pills on the CA1 cells in AD rats (HE staining, 200×). (B) The effect of Erzhi pills on neurons necrosis rate in hippocampal CA1 area of AD rats. (C) The effect of Erzhi pills on hippocampal CA1 region neurons of AD rats (Nissl staining, 200×). (D) The effect of Erzhi pills on the number of Nissl bodies in hippocampal CA1 area of AD rats. **^##^***p* < 0.01, vs. Sham-operated group; *p < 0.05, **p < 0.01, vs. model group. Mean ± SD, *n* = 6.

The results of the HE staining in the hippocampal CA1 area are shown in Figure 5(A,B). In the sham-operated group, the shape of observed neurons was round or oval, the cells were neatly arranged and without damage, with clear nucleoli and a large number of neurons. The model group showed a disordered arrangement of neurons, enlarged intercellular spaces with darker nuclear staining, fewer numbers of neurons, and a significantly increased neuronal death rate (*p* < 0.01). Compared with the model group, the oestradiol valerate and Erzhi pills groups had more neurons, with relatively complete morphology and in an orderly arrangement, and a significantly decreased neuronal death rate (*p* < 0.05), indicating that Erzhi pills and oestradiol valerate can protect hippocampal neurons.

The results of the Nissl staining in the hippocampal CA1 area are shown in Figure 5(C,D). In the sham-operated group, the structure of hippocampal neurons appeared normal, and the cytoplasm contained abundant Nissl bodies (86.50 ± 9.64). The model group showed reduced numbers of Nissl bodies in hippocampal neurons (58.50 ± 15.17) (*p* < 0.01) and a loss of neurons. Compared with the model group, the oestradiol valerate group (78.38 ± 10.23), Erzhi pills high-dose group (1.5 g/kg) (81.78 ± 13.51), and Erzhi pills low-dose group (0.75 g/kg) (72.67 ± 9.92) had more neurons, with relatively complete morphology and an increased number of Nissl bodies in the cytoplasm (*p* < 0.05).

### Effects of Erzhi pills on ERβ expression in the hippocampus of AD rats

**Figure 6.**
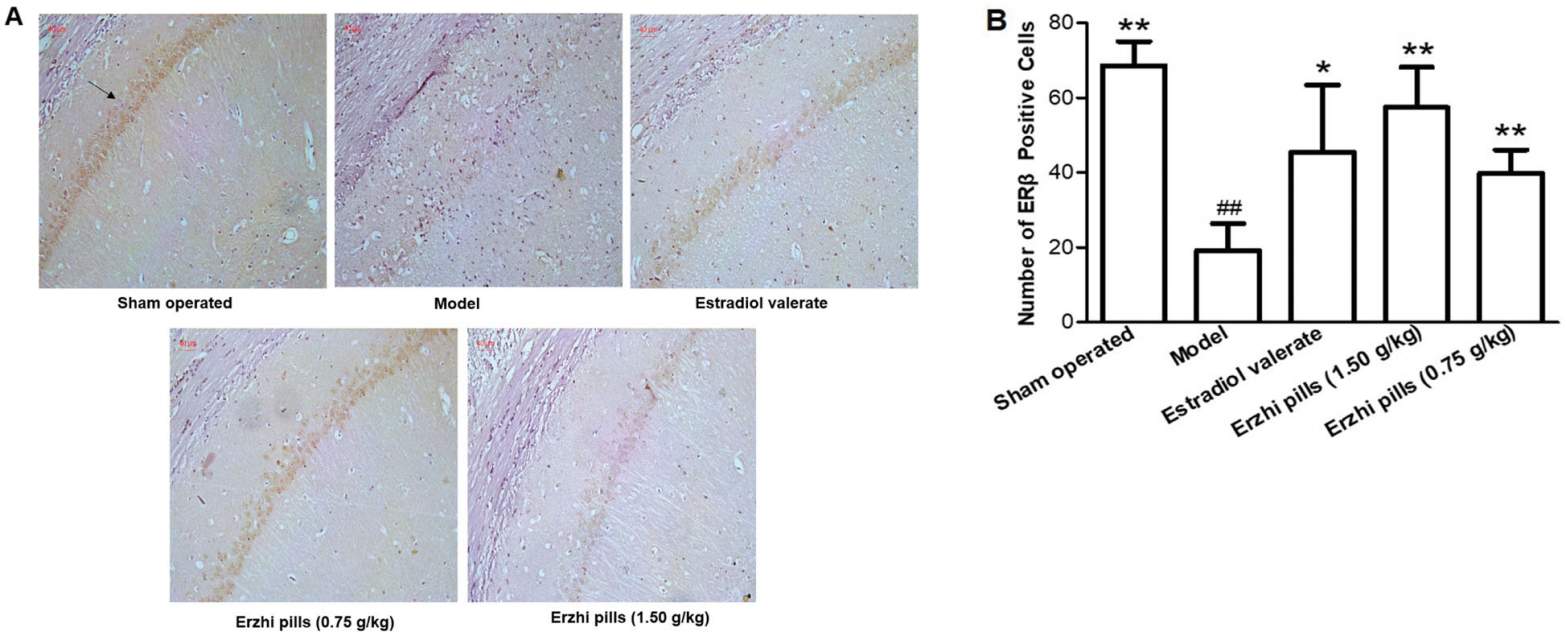
Effects of Erzhi pills on the expression of ERβ in hippocampus of AD model rats induced by ovariectomy as well as d-galactose and Aβ_1–40_ injection. (A) The effect of Erzhi pills on ERβ expression in hippocampus CA1 area of AD rats (IHC, 200×). (B) The effect of Erzhi pills on the number of ERβ positive cells in hippocampus CA1 area of AD rats. **^##^***p* < 0.01, vs. Sham-operated group; *p < 0.05, **p < 0.01, vs. model group. Mean ± SD, *n* = 6.

Results are shown in Figure 6(A,B). In the sham-operated group, the arrangement of neurons was more in order, they showed darker cytoplasm staining, and a large number of ERβ-positive cells (68.63 ± 6.47) was observed in the hippocampal CA1 area. In the model group, the arrangement of neurons was more disordered, cytoplasm staining appeared lighter, and the number of ERβ-positive cells was significantly reduced (19.14 ± 7.24) (*p* < 0.01). Compared with the model group, the oestradiol valerate group (45.57 ± 17.88), Erzhi pills high-dose group (1.5 g/kg) (57.42 ± 10.81), and Erzhi pills low-dose group (0.75 g/kg) (39.83 ± 6.24) had more Erβ-positive cells (*p* < 0.05), and neurons were neatly arranged with darker cytoplasm staining.

### *Effects of Erzhi pills on Aβ_1_*_–_ *_40_ and p-tau^404^ protein expression in the hippocampus of AD rats*

**Figure 7.**
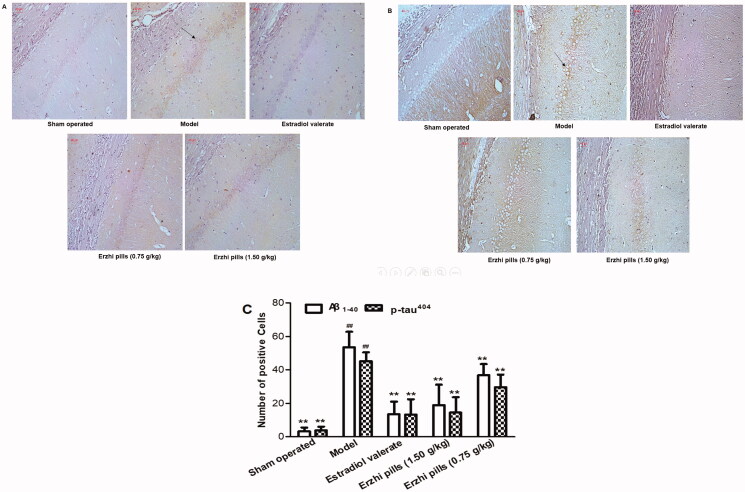
Effects of Erzhi pills on the expression of Aβ_1–40_ and p-tau^404^ in hippocampus of AD model rats induced by ovariectomy as well as d-galactose and Aβ_1–40_ injection. (A) The effect of Erzhi pills on Aβ_1–40_ expression in hippocampus CA1 area of AD rats (IHC, 200×). (B) The effect of Erzhi pills on p-tau^404^ expression in hippocampus CA1 area of AD rats (200×). (C) The expression of Aβ_1–40_ and p-tau^404^ Positive cells in hippocampus CA1 region of AD rats. **^##^***p* < 0.01, vs. Sham-operated group; **p < 0.01, vs. model group. Mean ± SD, *n* = 6.

The immunohistochemistry results of Aβ_1-40_ and p-tau^404^ in the hippocampal CA1 region are shown in Figure 7(A–C). In the sham-operated group, neurons were densely packed and had relatively shallow cytoplasm staining, pericellular staining was also lighter, while Aβ_1–40_-positive cells (3.14 ± 2.34) and p-tau^404^-positive cells (3.78 ± 2.22) were rarely seen in the hippocampal CA1 region. In the model group, the number of Aβ_1–40_-positive cells (53.50 ± 9.24) and p-tau^404^-positive cells (45.25 ± 5.28) was significantly increased (*p* < 0.01), neurons showed irregular shapes and a loose arrangement, the staining of the cytoplasm and pericellular space was darker; this suggests that the rat model of AD was established successfully. Compared with the model group, the number of Aβ_1–40_-positive cells (13.50 ± 7.45) and p-tau^404^-positive cells (13.14 ± 9.28) in the oestradiol valerate group, Aβ_1–40_-positive cells (18.85 ± 12.25) and p-tau^404^-positive cells (14.42 ± 9.18) in the Erzhi pills high-dose group (1.5 g/kg), and Aβ_1–40_-positive cells (36.83 ± 6.61) and p-tau^404^-positive cells (29.71 ± 7.43) in the Erzhi pills low-dose group (0.75 g/kg) were decreased (*p* < 0.01), while the staining of the cytoplasm and pericellular space was relatively shallow.

### Effect of Erzhi pills on hippocampal proteomics in AD rats

Differentially expressed proteins were screened by setting the parameter ratio to >1.5 or <0.5. Compared with the model group, 115 proteins were differentially expressed in the Erzhi pills groups. The classification of these 115 differentially expressed proteins was queried using the Uniprot database (https://www.uniprot.org/) according to protein ID; they included microtubule-related proteins, energy metabolism-related proteins, heat shock proteins, brain protection-related proteins, and Alzheimer’s disease-related proteins.

### Results of differential protein GO analysis

**Figure 8.**
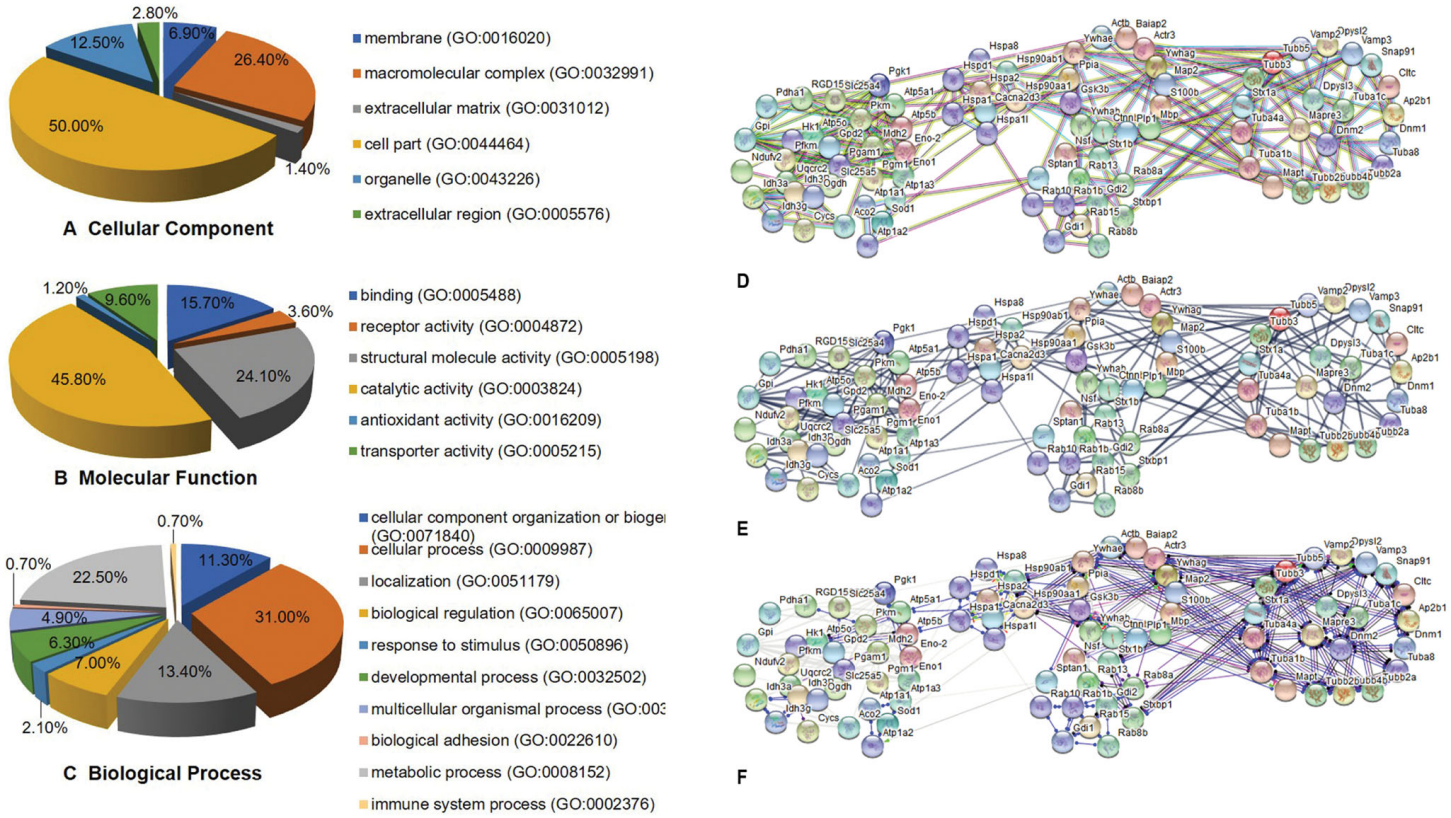
Results of differential proteins by GO analysis and String network analysis. (A) The Cellular Component analysis result of differential proteins. (B) The Molecular Function analysis result of differential proteins. (C) The Biological Process analysis result from differential proteins. (D) The evidence wiring diagram of protein. (E) The Confidence wiring diagram of protein. (F) The molecular action wiring diagram of protein.

The GO analysis results are shown in Figure 8(A–C). A total of 36 differential proteins were found to be distributed in cells; 19 are macromolecular complexes, nine are differential proteins distributed in organelles, five are differential proteins distributed in membranes, two are differential proteins distributed in extracellular regions, and one is a differential protein distributed in the extracellular matrix. Molecular functional analysis showed that most of the differentially expressed proteins had catalytic activity, followed by a structural molecular activity, binding activity, and transport activity. Forty-four differentially expressed proteins were found to be involved in cell processes, 32 in metabolic processes, 19 in localization, and 10 in biological regulation.

### String network analysis

Analysis of the STRING database found that 107 differential proteins were included in the STRING database and that these 107 proteins interact with each other. The signal network diagrams are shown in Figure 8(D–F). The top ten proteins in terms of the number of connections between proteins were RAC-α serine/threonine-protein kinase, actin, cytoplasmic 1, heat shock protein HSP 90-β, heat shock protein HSP 90-α, ATP synthase subunit α, glucose-6-phosphate isomerase, vesicle-associated membrane protein 2, 14-3-3 protein, glycerol-3-phosphate dehydrogenase, and Ras-related protein Rab-1B.

### Signal pathway enrichment analysis results in KEGG database

The differentially expressed proteins were imported into the KEGG database for signal pathway enrichment analysis. The results showed that these differential proteins are involved in 48 signalling pathways; six are involved in the synaptic vesicle cycle and Hippo signalling pathways, and five are involved in the PI3K-Akt signalling pathway, Huntington's disease, and Epstein-Barr virus infection (Table 2).

**Table 2.** The results of KEGG database signal path enrichment analysis (top 25).

Pathway	Count	Gene of participated protein
Synaptic vesicle cycle	6	Ap2b1, Cltc, Stx1a, Stx1b, Stxbp1, Vamp2
Hippo signalling pathway	6	Actb, Ctnnb1, Gsk3b, Ywhab, Ywhae, Ywhag
PI3K-Akt signalling pathway	5	Gsk3b, Ywhab, Ywhae, Ywhag, Akt1
Huntington’s disease	5	Ap2b1, Cltc, Ndufv2, Slc25a4, Sod1
Epstein–Barr virus infection	5	Gsk3b, Hspa1, Ywhab, Ywhae, Ywhag
Carbon metabolism	4	Aco2, Gpi, Mdh2, Ogdh
Metabolic pathways	4	Aco2, Gpi, Ndufv2, Ogdh
SNARE interactions in vesicular transport	4	Stx1a, Stx1b, Vamp2, Vamp3
Cell cycle	4	Gsk3b, Ywhab, Ywhae, Ywhag
Wnt signalling pathway	3	Gsk3b, CaMKII, β-catenin
MAPK signalling pathway	3	Cacna2d3, Hspa1, Mapt
Microbial metabolism in diverse environments	3	Gpi, Mdh2, Ogdh
Thyroid hormone signalling pathway	3	Actb, Ctnnb1, Gsk3b
Bacterial invasion of epithelial cells	3	Actb, Cltc, Ctnnb1
Influenza A	3	Actb, Gsk3b, Hspa1
AMPK signalling pathway	3	Rab10, Rab2a, Rab8a
Adherens junction	3	Actb, Baiap2, Ctnnb1
Arrhythmogenic right ventricular cardiomyopathy (ARVC)	3	Actb, Cacna2d3, Ctnnb1
Citrate cycle (TCA cycle)	2	Mdh2, Ogdh
Phagosome	2	Actb, Vamp3
Endocrine and other factor-regulated calcium reabsorption	2	Ap2b1, Cltc
Legionellosis	2	Hspa1, Rab1b
Parkinson s disease	2	Ndufv2, Slc25a4
Endocytosis	2	Ap2b1, Cltc
Alzheimer s disease	2	Gsk3b, Mapt

### Effects of Erzhi pills on PI3K/Akt signalling pathway in hippocampal tissues of AD rats

**Figure 9.**
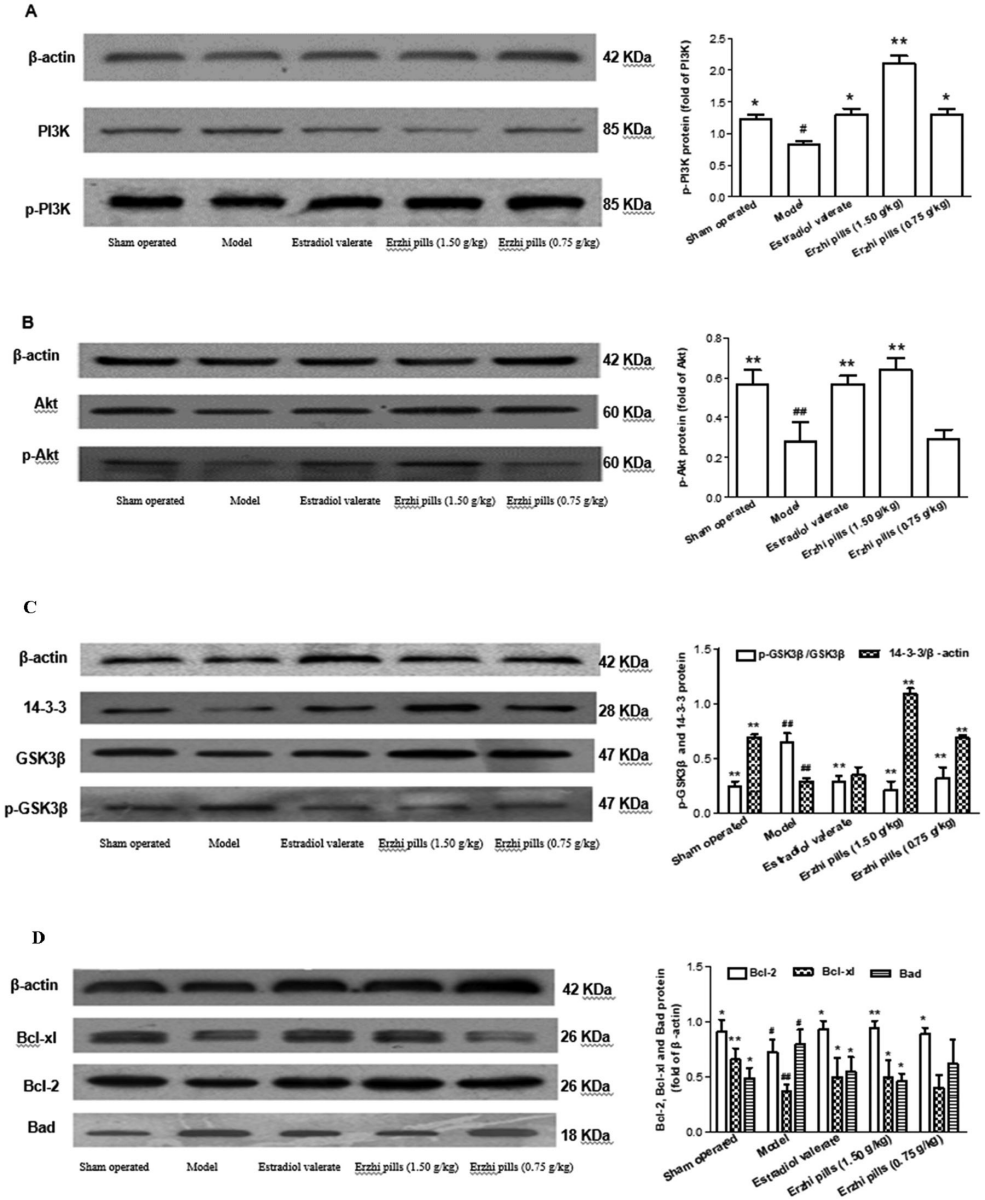
Effects of Erzhi pills on PI3K/Akt signalling pathway in hippocampal tissues of AD rats. (A) Erzhi pills enhance PI3K phosphorylation in hippocampal tissues of AD model rats induced by ovariectomy as well as d-galactose and Aβ_1–40_ injection. (B) Erzhi pills enhance Akt phosphorylation in hippocampal tissues of AD model rats induced by ovariectomy as well as d-galactose and Aβ_1–40_ injection. (C) Erzhi pills up-regulated 14-3-3 protein expressions and down-regulated GSK3β phosphorylation in hippocampal tissues of AD model rats induced by ovariectomy as well as d-galactose and Aβ_1–40_ injection. (D) Erzhi pills up-regulated Bcl-2, Bcl-xl protein expressions and down-regulated Bad protein expressions in hippocampal tissues of AD model rats induced by ovariectomy as well as d-galactose and Aβ_1–40_ injection. Data are presented as the mean ± SD. for at least five individual experiments. **^#^***p*＜0.05, **^##^***p*＜0.01, vs. Sham-operated group; *p < 0.05, **p＜0.01, vs. model group.

Results are presented in Figure 9. Compared with the sham-operated group, the expression of p-PI3K/PI3K, p-Akt/Akt, 14-3-3, Bcl-2, and Bcl-xl was significantly decreased (*p* < 0.05), while p-GSK3β/GSK3β and Bad expression was markedly upregulated in the model group (*p <* 0.05). Compared with the model group, the expression of p-PI3K/PI3K, p-Akt/Akt, 14-3-3, Bcl-2, and Bcl-xl was significantly increased (*p* < 0.05), p-GSK3β/GSK3β, while Bad expression was markedly downregulated in the oestradiol valerate and Erzhi pills high-dose groups (*p* < 0.05).

## Discussion

The pathogenesis of AD has not been fully elucidated, due to the long disease course and complex pathogenesis. The hallmark pathologies of AD are extracellular aggregates of Aβ plaques and intracellular aggregation of neurofibrillary tangles (NFTs), which are composed of hyperphosphorylated tau protein (Goedert 2015). These changes are eventually accompanied by neuronal damage and death (Patterson 2018). As there the world population is ageing, the incidence of AD is increasing, which places a serious burden on societies. Since 1998, only four drugs have been authorized as treatments of AD (Aricept, Exelon, galanthamine, and memantine). These drugs can suppress symptoms and delay the disease course, but they cannot cure AD (Patterson 2018). Therefore, it is necessary to explore further potential drugs for the treatment of AD. In this study, we demonstrate that Erzhi pills can improve serum levels of oestrogen, regulate the PI3K/AKT signalling pathway in the hippocampus, reduce Aβ deposition in the brain, inhibit the over-phosphorylation of Tau protein, maintain the morphology of hippocampal neurons, and attenuate the apoptosis of hippocampal neurons, thereby improving learning, memory, and cognitive function, in AD rats.

Numerous clinical studies have shown cognitive impairments in women following menopause or ovariectomy, which can be ameliorated by oestrogen-based hormone therapy (Sherwin 2009). The biological effects of oestrogens are mainly mediated by oestrogen receptor α (ERα) and oestrogen receptor β (ERβ). ERβ levels are higher than ERα levels in human and rat hippocampi (Bean et al. 2014). ERβ gene variations may increase the body’s susceptibility to AD and are strongly associated with the risk of AD in females (Pirskanen et al. 2005). Furthermore, oestrogens have been found to delay the apoptosis of nerve cells (Peri 2016). Oestrogen is closely associated with Alzheimer’s disease (AD). The model of senile dementia by ovariectomy is characterized by easy operation, low cost, and better stimulation of perimenstrual symptoms; therefore, it has been widely used in pharmacological and pharmacodynamic studies in recent years (Ibrahim et al. 2016). d-Galactose can decrease the growth and reproduction ability of cells and simulate oxidative damage in the brain, dysfunction of the cholinergic system, damage of hippocampal and cortical neurons, and attenuation of the expression of neurotrophic factors (Hua et al. 2007). Moreover, the animal model of AD induced by d-galactose combined with Aβ_1–40_ can better simulate the pathological features and processes of AD than the models induced by chemical or physical damage alone. Therefore, in the current study, a combination of ovariectomy, d-galactose, and Aβ_1–40_ was adopted to establish an AD model in rats (Chen et al. 2019). In this study, compared with the sham-operated group, the rats in the model group exhibited longer latencies on the MWM from day 2 of the training phase, and the number of platform crossings decreased significantly in the probe trial. In addition, the arrangement of hippocampal neurons in these rats was disordered, with larger intercellular spaces, more nuclear pyknosis, an increased neuronal necrosis rate, and fewer Nissl bodies due to loss of neurons, while oestrogen levels decreased and numbers of Aβ_1–40_- and p-tau^404^-positive cells increased significantly. These behavioural, morphological, and pathological experimental results suggest that we successfully established the AD rat model induced by ovariectomy combined with d-galactose and Aβ_1-40_ injection.

The MWM is an important method for assessing the learning, memory, and cognitive functions of animals, and is characterized by simple operation, sensitive detection, and high efficiency (Vorhees and Williams 2006). The pathological features of AD include a decreased number of pyramidal cells in the brain, a disordered and sparse arrangement of neurons, nuclear pyknosis, and a reduced cytoplasmic and nuclear ratio. The severity of AD can be determined using tissue slice staining (Walton 2012). Nissl staining is one of the most commonly used special staining techniques in the nervous system, which can reflect the function of cell metabolism by observing the number of Nissl bodies in neurons. Erzhi pills contain terpenes, flavonoids, lignans, and other compounds (all belonging to phytoestrogens), which can give rise to oestrogenic effects by binding to oestrogen receptors (Zhao et al. 2017; Fu et al. 2019). In the current study, we found that rats in the Erzhi pills groups had significantly shorter escape latencies on the MWM and higher numbers of platform crossings, and showed more orderly neuron arrangements and less nuclear pyknosis compared to rats in the model group. In rats treated with Erzhi pills, numbers of neurons, Nissl bodies, oestrogen levels, and ERβ-positive cells were increased, while numbers of Aβ_1–40_- and p-tau^404^-positive cells decreased. These results suggest that Erzhi pills can improve oestrogen levels, which may induce the upregulated expression of ERβ in hippocampal tissue, reduce the apoptosis of nerve cells, and further improve the learning and memory abilities of AD rats induced by ovariectomy combined with d-galactose and Aβ_1–40_ injection. In addition, Erzhi pills may relieve some of the cerebral pathological changes in AD model rats by reducing the loss of neurons and Nissl bodies, thereby improving learning and memory abilities and relieving cognitive dysfunction.

Proteomics is further used to evaluate the efficacy of traditional Chinese medicine by screening proteins with a differential expression before and after treatment. In our study, hippocampal proteomics was used as a starting point, targeting the key pathological site of AD, followed by an exploration of the molecular mechanism of Erzhi pills in preventing AD by analyzing the changes in different proteins. We identified more than 100 differentially expressed proteins between the Erzhi pills group and the model group, which are involved in 48 signalling pathways. In addition, five types of proteins are involved in the PI3K/Akt signalling pathway (14-3-3, GSK3β, Akt, Ywhae, and Ywhag). As the downstream protein of the PI3K/Akt signalling pathway, the content of 14-3-3 protein was significantly increased. Based on the correlation with AD, the number of different proteins involved in the pathway, and the significant changes in protein expression, we speculate that Erzhi pills can regulate the PI3K/Akt signalling pathway by upregulating 14-3-3 protein expression, subsequently decreasing phosphorylation of tau and inducing neuronal apoptosis and ultimately relieving the pathological symptoms of AD.

The PI3K/Akt signalling pathway plays an important role in maintaining homeostasis throughout the life cycle. This pathway is triggered by the expression or abnormal regulation of many genes and is involved in a variety of human diseases (Song et al. 2011). PI3K/Akt regulates signal transduction and biological processes, such as cell proliferation, apoptosis, and metabolism, and is involved in physiological processes of the central nervous system, such as cell survival, autophagy, neurogenesis, neuronal proliferation and differentiation, and synaptic plasticity (Long et al. 2021). PI3K/Akt performs a variety of biological functions through the phosphorylation or formation of downstream molecular complexes, such as FOXO, GSK-3β, mTOR, and actin-related proteins (Matsuo et al. 2018). The activation of p-Akt by Bad, GSK-3β, 14-3-3, and NF-κB can promote cell proliferation and inhibit cell apoptosis (Song et al. 2011). Upregulated expression of p-Akt/Akt can improve the memory capacity of AD rats (Li et al. 2016). In addition, activation of the PI3K/Akt pathway can inhibit neuronal apoptosis induced by Aβ aggregation (Quiroz-Baez et al. 2011). Studies have shown that activation of the PI3K/Akt pathway could alleviate behavioural symptoms and pathological progression in AD rats (Zhang et al. 2016). GSK-3 is a key regulator of axonal transport, cholinergic function, and synaptic plasticity, and GSK-3β has many substrates in neurons. Tau protein is the protein substrate responsible for the action of GSK-3β. Thus, GSK-3β and Tau are key targets in the pathogenesis of AD (Sayas and Ávila 2021). The 14-3-3 proteins are a family of proteins that are expressed throughout the body and have been implicated in many diseases, including cancer and neurodegenerative diseases. Low levels of 14-3-3 contribute to spinal loss and cognitive decline in AD (Pair and Yacoubian 2021). 14-3-3 has been shown to enhance the affinity of GSK3β to tau, thereby inhibiting the abnormal phosphorylation of tau protein (Sadik et al. 2009). In addition, 14-3-3 inhibits neuronal apoptosis (Shimada et al. 2013). Our proteomics results were subsequently validated by western blotting and suggest that Erzhi pills may improve the learning and memory abilities of AD rats by activating the PI3K/Akt signalling pathway via the upregulation of p-Akt, 14-3-3, Bcl-xl, and Bcl-2 and downregulation of p-GSK3β and Bad protein expression. In brief, the PI3K/Akt signalling pathway is involved in the pathogenesis of AD induced by ovariectomy combined with d-galactose and Aβ_1–40_ injection.

In summary, Erzhi pills ameliorate cognitive dysfunction and alter pathological features of the hippocampus by regulating oestrogen levels and proteomic profiles and then activating the PI3K/Akt signalling pathway in AD rats induced by ovariectomy combined with d-galactose and Aβ_1-40_ injection. The effects of Erzhi pills may be attributed to the interaction of its specific components. This study provides an important experimental basis for the clinical application of Erzhi pills in the prevention and treatment of AD.

## Conclusions

Erzhi pills can improve oestrogen levels, alter proteomic expression in the hippocampus, activate the PI3K/Akt pathway in AD rats, reduce Aβ aggregation, inhibit the hyperphosphorylation of Tau protein, maintain the morphology of hippocampal neurons, and attenuate the apoptosis of hippocampal neurons, thereby improving the learning and memory abilities of ovariectomized AD rats induced by d-galactose and Aβ_1–40_ injection. However, the active ingredients and specific targets of Erzhi pills in the prevention and treatment of AD rats have not been clearly defined and deserve further investigation.
